# Tolerance of Tumor-Specific T cells in Melanoma Metastases

**DOI:** 10.4172/2155-9899.1000409

**Published:** 2016-04-14

**Authors:** Katherine A Waugh, Sonia M Leach, Jill E Slansky

**Affiliations:** 1 University of Colorado School of Medicine, 12800 East 19th Avenue, Mail Stop 8333, Aurora, CO 80045, USA; 2 Center for Genes, Environment and Health, National Jewish Health, Denver, CO 80206, USA

**Keywords:** Tumor-infiltrating CD8+ T cells, T cell dysfunction, T cell hypofunction, Tolerance, Exhaustion

Compelling evidence associates functional CD8+ tumor-infiltrating lymphocytes (TIL) with increased survival of cancer patients [1]. However, tumors evolve to suppress T cells; TIL typically have a hypofunctional phenotype incapable of tumor clearance [2]. Exhaustion and tolerance are states of CD8+ T cell hypofunction that have been associated with TIL [1]. Many immunotherapies seek to restore or maintain antitumor function of CD8+ T cells [1]. As the mechanisms that restrict TIL function are elucidated, defining overlapping and exclusive pathways that restrict T cell function may accelerate identification of novel therapeutic targets.

T cell exhaustion is generally defined by accumulation of inhibitory receptors and gradual loss of functional capacities in response to persistent antigen stimulation and chronic inflammation [3,4]. Although exhausted CD8+ T cells have been most heavily characterized during chronic viral infections [5], similar states of exhaustion in TIL have been repeatedly documented in both murine and human tumors through phenotypic and functional characterization [3,4]. T cell tolerance controls inappropriate responses to self-antigens [3]. Many tumor-associated antigens recognized by CD8+ T cells are aberrantly expressed self-antigens [1,4]. Upon T cell receptor (TCR) stimulation, T cells lacking co-stimulation may undergo deletion or follow a transcriptional program toward a hypofunctional state of self-tolerance [3,6]. Self-tolerance characterization also includes loss of functional capacities and increased inhibitory receptor expression in some studies [1,6].

A pivotal genome-wide mRNA expression profile by Baitsch et al. of tumor-specific CD8+ T cells isolated from lymph node metastases (TILN) of vaccinated melanoma patients [4] showed that a gene set corresponding to exhausted CD8+ T cells [5] was enriched in TILN compared to functional counterparts [Gene set enrichment analysis (GSEA) [7,8]] (Figure 1). These results are not surprising because chronic inflammation and TCR stimulation are driving forces of TIL hypofunction during tumor growth [1]. Their supporting data showed TILN hypofunction and inhibitory receptor expression [4]. TILN were also compared to choice genes associated with deletional tolerance [9], but a genome-wide mRNA expression profile of self-tolerant CD8+ T cells was not yet available for systematic comparisons. Baitsch et al. therefore, concluded that exhaustion likely contributes to TILN hypofunction, but does not exclude the involvement of other transcriptional programs, such as self-tolerance [4]. Nevertheless, it has since become common for current literature to define hypofunctional TIL that express inhibitory receptors as “exhausted” [10,11].

Since a genome-wide mRNA expression profile of self-tolerant CD8+ T cells was recently made available by Schietinger et al. [6], we simultaneously analyzed TILN [4] for enrichment of genes associated with exhaustion [5] and self-tolerance [6] (Figure 2). We obtained statistics that adjust significance estimates for multiple hypothesis testing [7,8] (both self-tolerance and exhaustion FDR-corrected p-values <0.001). To our knowledge, these data are novel in that tumor-specific CD8+ T cells have not previously been systematically compared to self-tolerant CD8+ T cells. In light of the self-tolerant GSEA, the molecular and functional characterization by Baitsch et al. supports the conclusion that TILN [4] are exhausted and self-tolerant.

Because exhausted and self-tolerant CD8+ T cells share decreased functional capacity to proliferate in response to cognate antigen, it is of note that Schietinger et al. found the tolerance gene set to be associated with control of cell cycle (Figure 2B) [3]. Genes associated with cell cycle were also enriched among TILN and virally exhausted T cells when compared to their functional counterparts [4,5]. Enrichment of both gene sets in TILN further blurs the line between these states of hypofunctional CD8+ T cells. As T cell hypofunction varies by patient, malignancy, and over time, exhaustion and self-tolerance are not yet distinguishable by specific biomarkers [3]. These data show that unique non-overlapping gene expression defines TILN as heterogeneously exhausted and self-tolerant or as a distinct T cell program that partially overlaps with these T cell subsets (Table 1).

Nevertheless, a shift in focus towards overlapping mechanisms that underlie exhaustion and self-tolerance may benefit those that seek to elicit a functional response by TIL [1]. For instance, inhibitory receptor LAG-3 and co-stimulatory receptor 4-1BB have been studied in the context of viral exhaustion, self-tolerance, and TIL hypofunction [5,12–15]. These GSEA data suggest that therapies against LAG-3 [16] and 4-1BB [14,15,17] may release tumor-specific CD8+ T cells from exhaustion and self-tolerance (Table 1).

Both viral-exhaustion and self-tolerance can be temporarily overridden to promote functional T cell responses [3]. Exhaustion occurs gradually upon chronic non-self TCR or immunostimulatory signals, whereas self-tolerance generally occurs quickly in response to initial TCR exposure to self-antigen when additional immunostimulatory signals are lacking [3]. Dissection of the overlapping and distinct gene expression between a tumor-specific CD8+ T cell program of hypofunction, exhaustion, and self-tolerance has widespread implications for development of cancer immunotherapies to mobilize TIL against both non-mutated self or mutated tumor antigens.

## Figures and Tables

**Figure 1:**
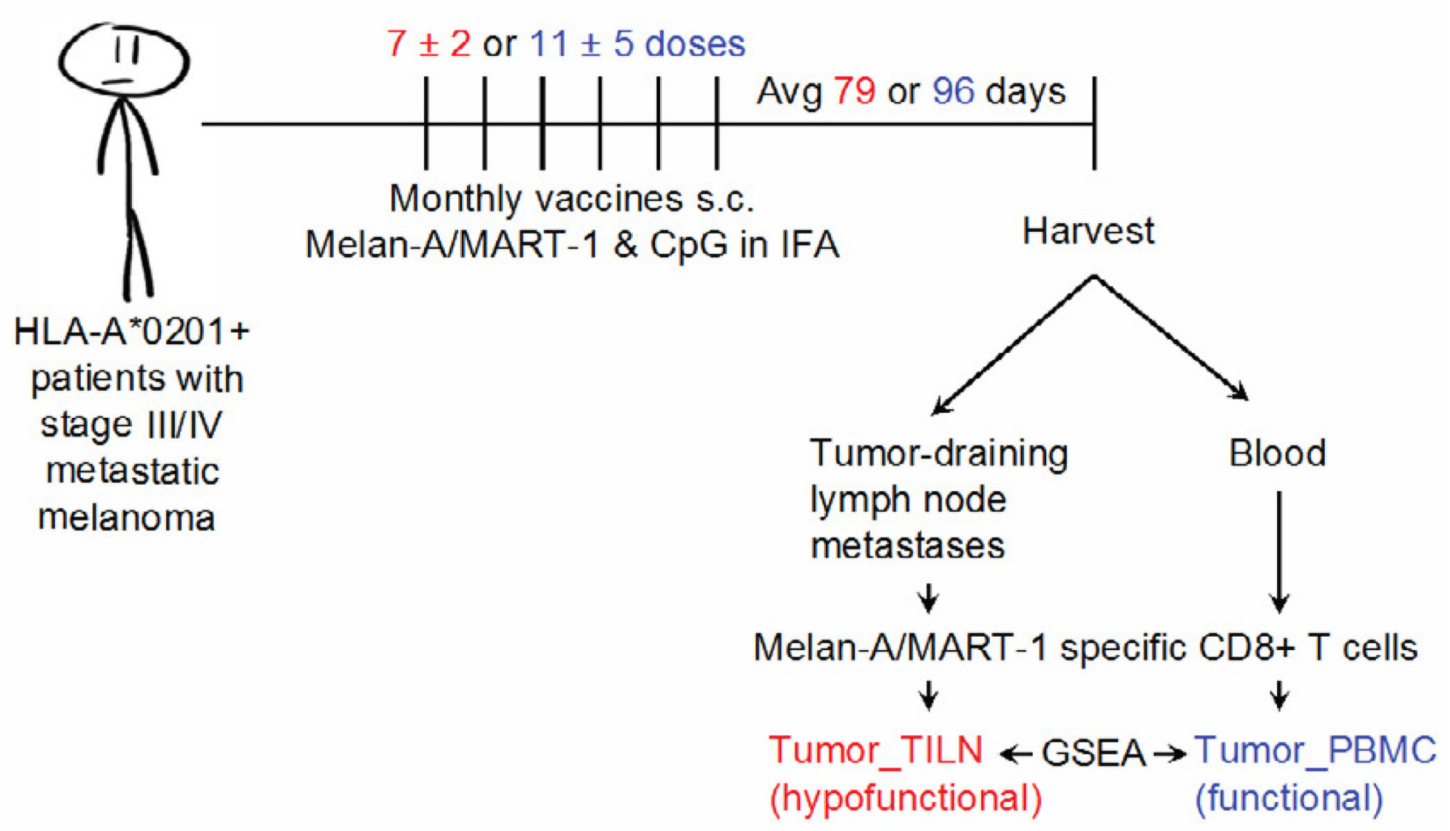
Tumor-specific CD8+ T cells were expanded by vaccinations and isolated for genome-wide mRNA expression profiling, as described [4]. Briefly, tumor metastases (red) and peripheral blood (blue) samples were obtained from cancer patients with stage III or IV melanoma and an HLA-A*0201 allele. Patients had received monthly subcutaneous (s.c.) vaccinations of peptide Melan-A/MART-1 and adjuvant CpG emulsified in incomplete Freund’s adjuvant (IFA). Number of vaccinations varied by 2 or 5 doses. “Avg” represents the average number of days from final vaccine boost until T cell analyses. Genome-wide mRNA expression was determined by microarray (Agilent) of live CD8+ T cells FAC-sorted on HLA-A2: Melan-A/MART-1 tetramer [4]. Differential gene expression between Melan-A/MART-1 specific CD8+ T cells from the tumor (TILN_hypofunctional) and blood (Tumor_PBMC) were systematically compared to previously published gene expression profiles of other CD8+ T cell subsets [5] by gene set enrichment analysis (GSEA) [4,7,8].

**Figure 2:**
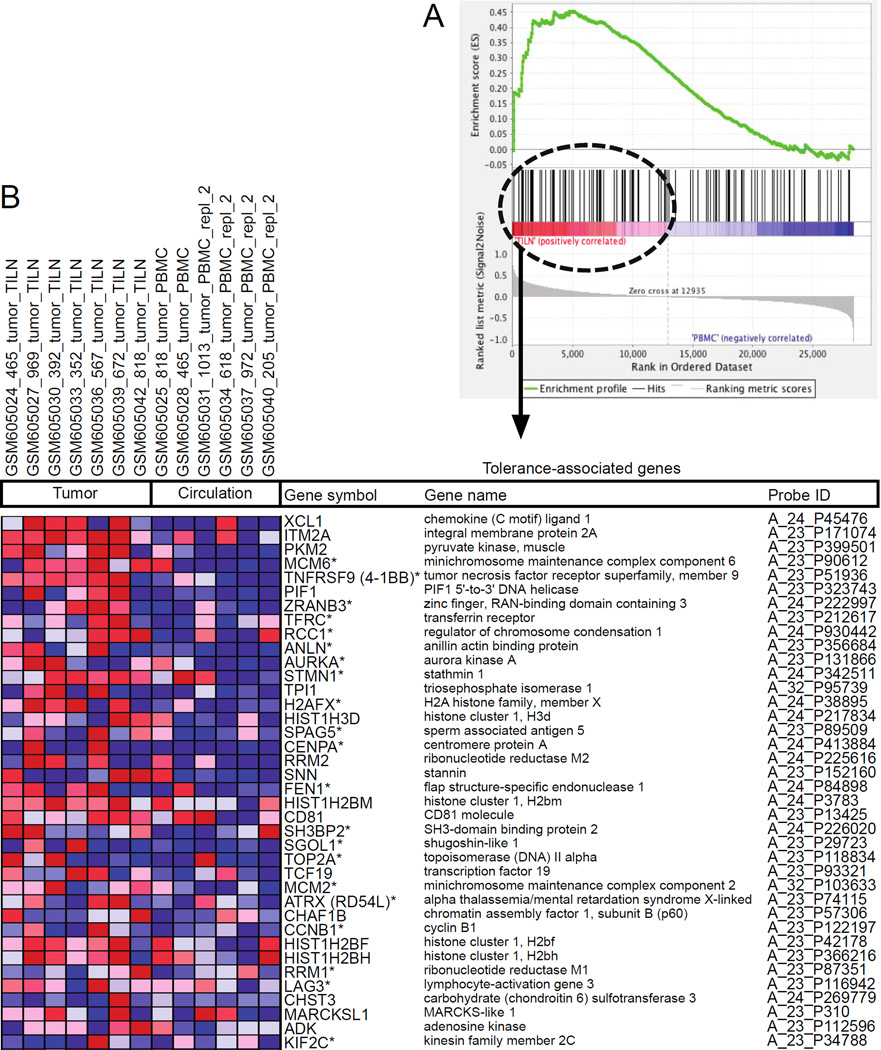
Gene set enrichment analysis (GSEA) of genes associated with self-tolerance in tumor-specific CD8+ T cells. Expression data of TILN [4] were analyzed for genes associated with self-tolerance [6] by GSEA using parameters recommended for expression datasets that contain a sample with less than 7 replicates: dataset and gene sets were converted into gene symbols, redundant probe sets were collapsed using probe medians, a Signal2Noise metric was used for ranking genes, and the weighted enrichment statistic and 1000 gene set permutations were employed [7,8]. The 144 genes associated with self-tolerance were previously published in K-means clusters 9 and 13 [6]. We converted 119 of these mouse genes to human homologues in DAVID [18,19]; before comparing them to genes expressed by tumorspecific CD8+ T cells [4,7,8]. (A) The plot shows enrichment of genes associated with tolerance in TILN compared to tumor-specific CD8+ T cells in circulation. The Normalized Enrichment Score (1.64, green line) considers the ranked list of expression differences between tumor-specific CD8+ T cells from the tumor and periphery (red=increased in TILN, blue=decreased in TILN). Vertical black lines indicate where genes overexpressed by self-tolerant versus functional T cells [6] fall in the ranked list [4] and significantly cluster among genes most expressed by TILN (p-value <0.001 and FDR <0.001) [7,8]. (B) The 39 genes that comprise the leading edge of the Enrichment Score [7,8] are shown in a corresponding heat map. The color gradient matches the location of genes associated with self-tolerance among the ranked list of gene expression by tumor-specific CD8+ T cells (red=increased in TILN, blue=decreased in TILN). Genes denoted with an asterisk are associated with the cell cycle (p-values=1.47 × 10^−2^ – 6.37 × 10^−11^) through the use of QIAGEN’s Ingenuity^®^ Pathway Analysis (IPA^®^, QIAGEN, Redwood City, CA, USA, www.qiagen.com/ingenuity).

**Table 1:** Distinct core genes drive TILN enrichment of tolerance and exhaustion gene sets. Core genes differ in the leading edges of the exhaustion [5] and tolerance [6] gene sets compared to the TILN versus PBMC analysis [4]. Leading edge genes (circled in Figure 2A) are the core genes that drive the enrichment score, which is statistically significant for both the exhaustion and tolerance comparisons.

Exhaustion (78)					Self-tolerance (35)				Shared (4)
ACADM	CIT	HSPA8	MDFIC	PLK4	SH3BGRL	ADK	HIST1H2BN	RRM2	CCNB1
ASCC1	E2F8	IDH2	MKI67	PON2	SNRPB2	ANLN	HIST1H3D	SGOL1	LAG3
ATF1	EIF2S1	IFNG	NDFIP1	PRKD2	SS18	AURKA	ITM2A	SNN	TNFRSF9 (4-1BB)
CCL4	ELL2	IL6ST	NFIL3	PTPN6	SUPT4H1	CD81	KIF11	SPAG5	TOP2A
CCND2	ENG	IRF4	NFKBIZ	RERE	TACC3	CENPA	KIF2C	TCF19	
CCR5	ENTPD1	ISG20	NR4A2	RNF11	TCF7	CHAF1B	MARCKSL1	TFRC	
CCT8	EVL	ITGA4	NRP1	RPA2	TNFRSF1B	CHST3	MCM2	TPI1	
CD160	FAM102A	ITGAE	NUCB1	RSAD2	TTC3	FEN1	MCM6	XCL1	
CD244	FAM134B	JAK3	NXF1	SDHA	UBR4	H2AFX	PIF1	ZRANB3	
CD7	FOS	KLF10	OSBPL11	SELL	VAMP7	HIST1H2BF	PKM2		
CD9	FYN	KPNB1	PBX3	SERPINB9	WNK1	HIST1H2BH	RAD54L		
CHEK1	GZMK	LBR	PDK1	SFMBT2	ZFP36	HIST1H2BJ	RCC1		
CIRH1A	HMGCS1	LCLAT1	PELI1	SGK1	ZFP91	HIST1H2BM	RRM1		
